# Amino acids in hematologic malignancies: Current status and future perspective

**DOI:** 10.3389/fnut.2023.1113228

**Published:** 2023-03-23

**Authors:** Mengyao Wang, Ailin Zhao, Meng Li, Ting Niu

**Affiliations:** Department of Hematology, West China Hospital, Sichuan University, Chengdu, Sichuan, China

**Keywords:** amino acid, hematologic malignancy, metabolism, therapy, glutaminolysis, glutathione

## Abstract

In recent years, growing emphasis has been placed on amino acids and their role in hematologic malignancies. Cancer cell metabolism is altered during tumorigenesis and development to meet expanding energetic and biosynthetic demands. Amino acids not only act as energy-supplying substances, but also play a vital role *via* regulating key signaling pathways, modulating epigenetic factors and remodeling tumor microenvironment. Targeting amino acids may be an effective therapeutic approach to address the current therapeutic challenges. Here, we provide an updated overview of mechanisms by which amino acids facilitate tumor development and therapy resistance. We also summarize novel therapies targeting amino acids, focusing on recent advances in basic research and their potential clinical implications.

## 1. Introduction

**Figure 1:**
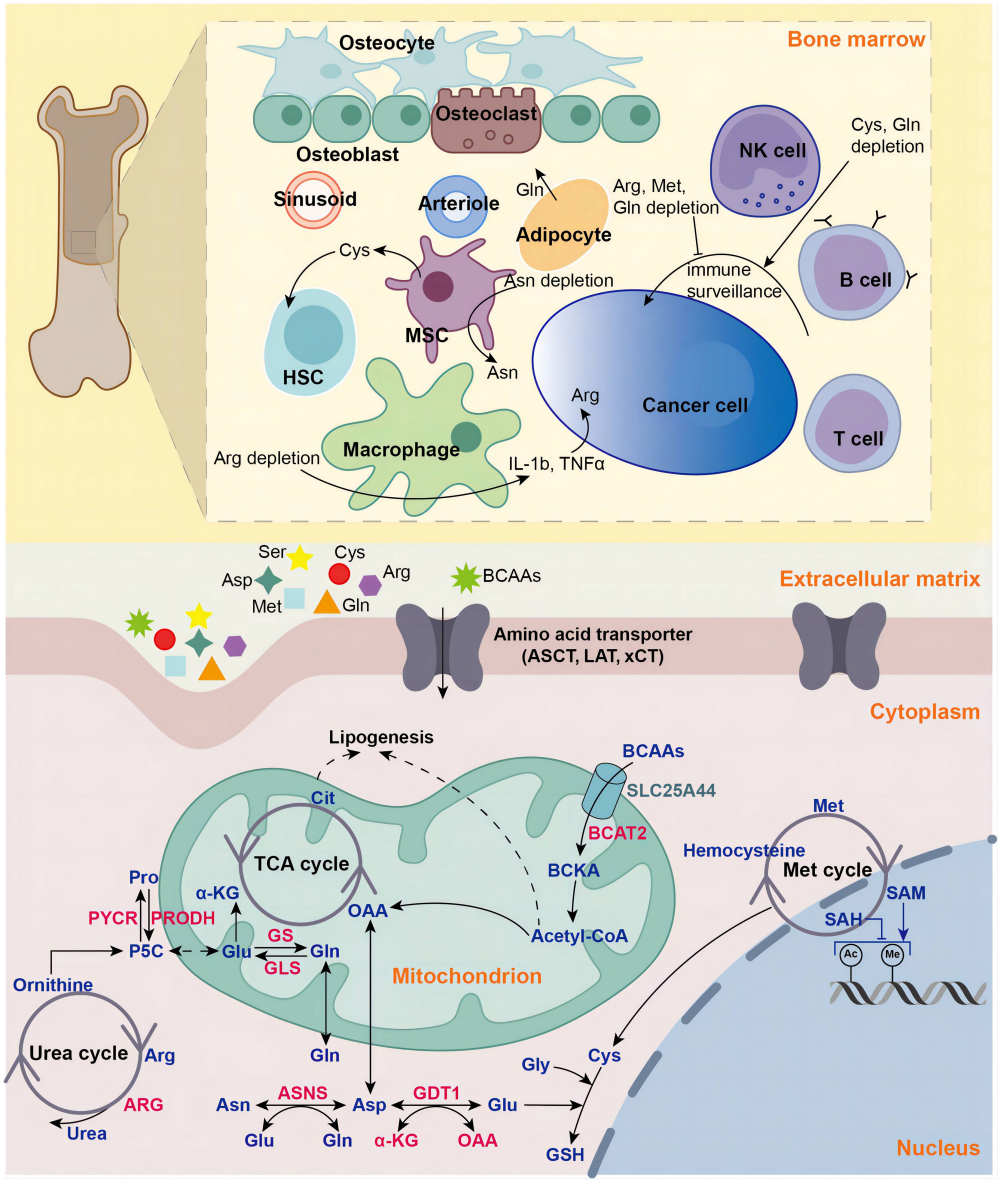
Amino acids in hematologic malignancies. Amino acids play vital roles in in tumorigenesis and development in hematologic malignancies, including energy supply, biosynthetic support, redox balance maintenance, epigenetic regulation and tumor microenvironment modulation. NK natural killer, MSC mesenchymal stromal cell, HSC hematopoietic stem cell, Gln glutamine, Glu glutamate, Gly glycine, Cys cysteine, Asp aspartate, Pro proline, Asp aspartate, Asn asparagine, Arg arginine, Met methionine, BCAA branched-chain amino acid, BCKA branched-chain ketoacid, aspartate transaminase (AST), GLS glutaminase, GS glutamine synthetase, ASNS asparagine synthetase, PRODH pyrroline-5-carboxylate dehydrogenase, PYCR pyrroline-5-carboxylate reductase, P5C pyrroline-5-carboxylate, ARG arginase, GSH glutathione, acetyl-coA acetyl-coenzyme A, α-KG alpha-ketoglutaric acid, OAA oxaloacetic acid, LAT large-neutral amino acid transporter, ASCT (alanine, serine, cysteine transporter), xCT cystine/glutamate antiporter, SLC25A44 solute carrier family 25 member 44, TCA tricarboxylic acid, Cit Citric Acid, SAM S-adenosylmethionine, SAH Sadenosyl homocysteine.

Uncontrollable cellular proliferation, invasion and metastasis are the characteristics of cancer cells (1, 2). To accommodate more rapid proliferation, tumor cells alter their metabolism to provide sufficient cellular structural substances (proteins, DNA, RNA, and lipids) and energy. Aberrant metabolism is also an important feature of cancer (2–4). The Warburg effect is the most well-known cancer-specific metabolic feature (2, 5). Cancer cells rewire their metabolic pathways, preferentially metabolizing glucose through aerobic glycolysis pathway to generate ATP efficiently, even under aerobic conditions (2). Besides glucose, cancer cells also develop increased reliance on amino acids (AAs) to meet enhanced demands for energy and cellular building blocks (6). Recently, increasing evidences show that AAs play vital roles in tumor development (6). AAs are not only involved in energy-generating and biosynthetic (4), but also exert cancer-promoting effects by regulating signaling pathways (7), epigenetic processes (8), cellular redox state (9) and immunosuppressive tumor microenvironment (TME) (10) (Figure 1).

Requirements for AAs is different between normal and tumor cells, which can be exploited to develop anti-cancer therapies. Multiple strategies targeting AA metabolism have been developed, including dietary AA starvation, AA depletion, inhibition of AA transporters and synthases (4). In clinical practice, the utilization of therapies targeting AA is more common in hematologic malignancies and some have achieved remarkable therapeutic outcomes (11). L-asparaginase is the most successful example of AA deprivation therapy, which has changed the landscape of acute lymphoblastic leukemia (ALL) treatment (12). Promising new drugs are continually emerging. These novel agents not only demonstrate potential efficacy in monotherapy, but also show good results in reversing drug resistance and relieving immunosuppression (6). In this review, we discuss mechanisms of action of AAs in hematologic malignancies, focusing on novel roles beyond rewired metabolism. We also summarize deprivation strategies using heterologous agents and recent data from clinical trials.

## 2. Amino acids in hematologic malignancies

### 2.1. Glutamine

Glutamine (Gln) is a nonessential AA. Intracellular glutamine mainly originates from the bloodstream (13). Gln is involved in a broad range of cellular activities, including energy supply, biosynthesis of other biomolecules and maintaining cellular redox homeostasis (14). Besides glucose, Gln is the most important energy supplyinig substance. As glucose is metabolized through anaerobic metabolism to produce lactate, Gln enters the tricarboxylic acid (TCA) cycle and provides TCA metabolites, supporting generation of lipids, proteins, and nucleic acids (13). Enhanced glutaminolysis is a prominent feature of various cancers (15).

Gln is the most abundant AA in serum and is avidly taken up by tumor cells (13). Gln is transported into cells and mitochondria through solute carrier (SLC) family members, mainly SLC1A5 (also known as alanine, serine, cysteine [Cys] transporter 2, ASCT2) (16, 17). In addition to transport, another important way for Gln entering cells is macropinocytosis. Macropinocytosis is a type of endocytosis in which extracellular fluid and nutrients are engulfed and taken up *via* large endocytic vesicles known as macropinosomes (18). L-type AA transporter 1 (LAT1), a heterodimer of SLC7A5 and SLC3A2, serves as an antiporter responsible for exporting Gln in exchange for leucine (19).

Gln dissociates into ammonium ions and glutamate under the catalysis of mitochondrial glutaminases (GLS) or cytoplasmic asparaginase (ASNase). Glutamate is then metabolized *via* two major pathways. Glutamate is converted into c-glutamylcysteine, which is utilized to generate glutathione (GSH) by GSH synthetase (13). By quenching reactive oxygen species and participating in the ascorbate-glutathione cycle, which eliminates peroxides, GSH protects both normal and malignant cells from oxidative injury. Leukemic cells have redox dysregulation, including aberrant GSH metabolism, thus making them sensitive to pro-oxidant therapies that further disrupt GSH pathways (20, 21).

When catalyzed by glutamate dehydrogenase (GDH), glutamate breaks into α-ketoglutarate (α-KG) and ammonia. When catalyzed by transaminases, glutamate transfers amino groups to generate other AAs (proline, aspartate, serine and alanine) and α-KG. α-KG produced by these two processes enters the TCA cycle, where it participates in mitochondrial oxidative phosphorylation (OXPHOS) and eventually generates ATP (15). Some hematologic tumor cells are addicted to Gln-fueled OXPHOS (9, 22). For example, in mantle cell lymphoma (MCL), lymphoma cells reprogram metabolically toward OXPHOS and glutaminolysis to gain advantage in generating energy and develop drug resistance to Bruton’s tyrosine kinase (BTK) inhibitors (9). Acute myeloid leukemia (AML) cells are sensitive to OXPHOS controlled by Gln (22). Interestingly, leukemia stem cells (LSCs) are characterized by a low rate of energy metabolism and a low cellular oxidative status. They are unable to utilize glycolysis and dependent on OXPHOS for energy generation (23). Furthermore, the isocitrate dehydrogenase (IDH) transforms isocitrate into α-KG. Mutated IDH, caused by *IDH1/2* mutations, transforms α-KG into 2-hydroxyglutarate (2-HG), which represses demethylases of both DNA and histones, leading to aberrantly increased methylation levels of both DNA and histones and epigenetic dysregulation (24). *IDH1* mutations and *IDH2* mutations have been reported in 6%–9% and 8%–12% AML patients, respectively (25, 26). In low-risk or medium-risk AML patients with normal karyotype, *IDH1*/2 mutations were significantly related to worse prognosis (27, 28).

Similar to glucose, cancer cells also increase uptake and utilization of Gln to meet energy requirement and biosynthetic demands of rapid cell growth. Gln transporters and enzymes involved in glutaminolysis are highly expressed in many hematological malignancies (15). SLC overexpression was observed in most leukemia, lymphoma and myeloma and associated with poor prognosis (29). For example, Bolzoni et al. discovered that SLC1A5 was highly expressed in multiple myeloma (MM) cells and was needed for MM growth (30). Myc or Ras-driven tumors were particularly dependent on exogenous Gln (31). In Burkitt lymphoma, MYC increased the expression of both SLC1A5 and SLC7A5, promoted glutaminolysis and led to augmented tumor proliferation (32). In natural-killer T-cell lymphomas (NKTCLs), SLC1A1 acted as a central regulator of aberrant Gln metabolism (33). SLC1A1 overexpression in lymphoma not only enhanced tumor growth, but also promoted competition for Gln between lymphoma cells and CD8^+^ T cells. Impaired CD8^+^ T cell activity, together with PD-L1 downregulation, led to immunosuppression and tumor progression (33).

Considering the close connection between malignancies and Gln metabolism, disrupting Gln utilization in tumor can be an important means to impede tumor growth. Current therapeutical strategies are mainly focused on hindering cancer cells from obtaining and utilizing Gln, comprising the following aspects: (1) Blocking Gln transporters, including SLC1A5, SLC7A11 and cystine/glutamate antiporter (xCT) system. (2) Inhibition of enzymes involved in Gln metabolism. (3) Gln depletion: ASNase degrades Gln as well as Arg, which will be discussed in detail in the section of Arg-based therapeutic approaches (15, 34, 35). These therapeutic agents have shown excellent results in preclinical studies and some have already entered clinical trials. In the following discussion, we focus mainly on agents that have exhibited promising efficacy in hematological malignancies.

γ-Glutamyl-p-nitroanilide (GPNA) and V9302 are ASCT2 inhibitors. Results from preclinical studies have shown that blocking ASCT2 with GPNA inhibits proliferation and induces apoptosis in AML cells, thus prolong survival in AML mice (36). Blockade of ASCT2 with V-9302 also leads to effective tumor control (37, 38). It is noteworthy that, besides abrogating glutaminolysis, observed efficacy of V-9302 may be due to combinatorial blockade of multiple ASCT2 substrates. Elevated autophagy, increased oxidative stress and decreased mTOR activity were observed in V-9302-treated cancer, which implies a theoretical basis for rational combination therapy (37).

Multiple drugs act on enzymes involved in Gln metabolism. GLS inhibitors include CB-839 (Telaglenastat), bis-2-(5-phenylacetamido-1,3,4-thiadiazol-2-yl)ethyl sulfide (BPTES) and compound 968. [6-diazo-5-oxo-L-nor-leucine (DON)] and JHU083 have structural similarities with Gln, thereby inhibiting all related enzymes (35, 39).

CB-839 is the most well-studied GLS inhibitor. CB-839 exhibited potent anticancer activity against a variety of tumors in preclinical studies. Anticancer effects were more remarkable when combined with agents of other mechanisms of action (35). Increased mitochondrial respiration was found in proteasome inhibitors (PIs)-resistant MM cells (40). Gln was the primary fuel for mitochondrial respiration, cell proliferation and survival in both PI-sensitive and PI-resistant MM cells. CB-839 significantly blocked mitochondrial respiration and demonstrated dramatic synergy with PIs (40). AML cells were highly dependent on the Gln for their survival. Inhibiting Gln metabolism *via* CB-839 significantly impaired antioxidant GSH production in multiple types of AML, leading to accumulation of mitochondrial reactive oxygen species (ROS) and apoptosis. Together with the pro-oxidant drug, such as arsenic trioxide (ATO) and homoharringtonine (HHT), CB-839 induced more mitochondrial oxidative stress and led to more thorough leukemic cell elimination (41). In *FLT3*-internal tandem duplication (*FLT3*-ITD)-positive AML, a subtype of leukemia with notoriously dismal outcome, CB-839 also impaired GSH production, induced severe mitochondrial oxidative stress and cell apoptosis. More remarkably, CB-839 and FLT3 inhibitors, including AC220 and gilteritinib, exerted synergistic pro-apoptotic effects and displayed more potent anti-leukemia effect (42, 43). These redox-targeted combinations represented a novel therapeutic strategy with high efficiency, low toxicity and enormous potential for clinical translation (44). At present, CB-839 has entered clinical trials for treatment of leukemia, myelodysplastic syndromes (MDS), renal cell carcinoma, and non-small cell lung cancer. A phase II study (NCT0347993) was conducted to evaluate safety and efficacy of CB-839 (600 mg BID orally) in combination with standard Azacitidine (Aza) in advanced MDS patients (14). Primary efficacy outcome showed that 62.5% (10/16) patients achieved marrow complete response (CR) and 31.3% (5/16) had stable disease (SD). Adverse events analysis showed that the most common non-hematological adverse events (AEs) were gastrointestinal abnormalities (62.5%), including nausea, constipation, elevated ALT and anorexia. The most frequent hematological AEs were grade III-IV anemia (12.5%), neutropenia (37.5%) and thrombocytopenia (37.5%). 43.7% patients experienced grade III-IV infections. This interim analysis demonstrated that the combination of CB-839 and Aza was a promising treatment with acceptable safety profile and encouraging response rates.

DON is a glutamine antagonist with robust anticancer efficacy. But its clinical use was hampered due to its severe toxicity, especially gastrointestinal toxicity and mucositis (45). In recent years, various prodrugs for DON have been synthesized to enhance delivery of active compound to tumor lesions (39, 46). This delivery strategy enhanced the anti-tumor effects of DON while circumventing its toxicity, bringing it back on track. JHU083 is a dual promoeity prodrug modified from DON. JHU083 possessed several advantages over DON: enhanced oral bioavailability, tumor-targetability and central nervous system (CNS) penetration (47).

### 2.2. Arginine

Arginine (Arg) is considered as a non-essential or semi-essential AA. The sources of Arg include dietary uptake, protein turnover and endogenous synthesis (48). Healthy individuals are capable of synthesizing Arg from Gln, glutamate, and proline. However, when the demand for Arg is increased, such as growth during infancy, pregnancy, and burn injuries, endogenous biosynthesis of Arg may be insufficient to support body needs, and dietary sources of Arg become essential (49). Arg is one of the most versatile AAs. Arg is involved in a variety of biochemical metabolic processes, such as the urea cycle and the TCA cycle (50). It also participates in regulating several important physiological activities, including cell proliferation, immunity and hormone secretion. Besides, Arg is the major precursor for synthesis of cancer-associated compounds such as polyamines and nitric oxide (NO) (50).

Normal cells synthesize Arg intracellularly from ornithine and citrulline under the catalysis of argininosuccinate synthase (ASS) 1, ornithine transcarbamylase (OTC), and argininosuccinate lyase (ASL). However, over 70% of cancers are deficient of these key enzymes, rendering them highly dependent on external Arg (51). The addiction of cancer cells for Arg is called Arg auxotrophism, Arg auxotrophism is a prominent feature of, hematologic malignancies which may be attributed to methylation of the ASS1 promoter (52, 53). For example, Mussai et al. identified that there was low or no expression of ASS and/or OTC in AML blasts. Bone marrow stroma may play a vital role in producing Arg to support AML expansion (51).

There are two major pathways of Arg catabolism involving four main enzymes: nitric oxide synthase (NOS), arginases (ARGs), arginine decarboxylase and arginine:glycine amidino transferase (OAT). One catabolic pathway is mediated by NOS, which catabolizes Arg into citrulline and NO (50). NO has two-way effects on tumor growth, depending on its concentration, time of exposure, cellular redox status and TME. In general, low concentration of NO promotes tumorigenesis and development, whereas high-level NO can cause DNA damage, induce apoptosis and activate immune defense (54). For example, in B-cell chronic lymphocytic leukemia (B-CLL), leukemic cells expressed inducible NOS and increased NO release. The anti-apoptotic effect of NO could be counteracted by NOS inhibitors and engagement of the APO-1/Fas pathway (54). However, exogenous supplementation of NO promoted cell death and potentiated the cytotoxic effect of fludarabine to B-CLL lymphocytes (55). ARGs compete with NOS to catabolize Arg decomposition. ARGs decompose Arg into urea and ornithine. Ornithine is an important intermediate. The vast majority of ornithine participates in the urea cycle: it is converted to citrulline by ornithine carbamoyltransferase (ODC) and subsequently involved in synthesis of Arg under the catalysis of ASS. Ornithine can also be converted into polyamines, which also promote cell proliferation and tumor growth (50).

Since most hematologic malignancies are auxotrophic for Arg, starving tumors by exhausting Arg is a potential therapeutic strategy (51). Two classes of Arg depleting agents, ARG and arginine deiminase (ADI), have achieved a certain level of success and moved into clinical trials (56, 57). PEG-ARG1 and BCT-100 are pegylated arginases. Rodriguez et al. found that in T-cell acute lymphoblastic leukemia (T-ALL), PEG-ARG1 triggered cell apoptosis and inhibited proliferation through phosphorylation of the eukaryotic-translation-initiation factor 2 alpha (eIF2a) (58). Combination of PEG-ARG1 with cytarabine (Ara-C) or phospho-eIF2a signaling significantly prolonged the survival of mice bearing T-ALL, representing a potential treatment therapy for this high-risk subtype of leukemia (59).

ADI, an enzyme derived from mycoplasma, rapidly degrades Arg intro citrulline (56, 57). To avoid adverse effects of foreign proteins, such as anaphylaxis and rapid clearance, ADI-PEG20, a pegylated form of ADI, has been developed. Results from preclinical studies showed that ADI-PEG 20 induced caspase-dependent cell apoptosis and autophagy in leukemia and lymphoma (50). ADI-PEG 20 depleted Arg and showed a significant killing effect on AML, which could be further enhanced when combined with cytarabine (60). In a phase II study (NCT01910012), single-agent ADI-PEG20 resulted in a moderate disease control rate (DCR) of 42.9% in relapsed/refractory/poor-risk AML patients (61). Its hematologic and non-hematologic toxicity was minimal when compared with chemotherapy.

### 2.3. Asparagine

Asparagine (Asn) is a multifunctional AA. It plays important roles in supporting cell proliferation and growth, especially in protein synthesis. Asn residues provide amide nitrogen that allows N-linked glycosylation, contributing to glycoprotein synthesis (62, 63). Intracellular Asn also acts as AA exchange factor. Asn is exported in exchange for extracellular AAs, especially serine, arginine and histidine, the process of which activates mTOR complex 1 (mTORC1) and promotes protein and nucleotide synthesis (64). Asn is also found to suppress apoptosis induced by Gln deprivation (65).

Asn is considered as a non-essential AA for normal human cells. Asn can be synthesized by Gln and aspartate under the catalysis of Asparagine synthetase (ASNS) in an ATP-dependent manner (65). ASNase hydrolyzes ASN into aspartate and ammonia. In some cancer cells, the expression of ASNS is low or even absent, rendering them unable to synthesize Asn by themselves. Once Asn from bloodstream is depleted, these cancer cells will suffer from Asn starvation and subsequently undergo apoptosis. Asn deprivation is an effective treatment strategy for cancers lack or deficient of ASNS expression, including hematological malignancies (12).

L-asparaginase (L-ASNase) is isolated from *Escherichia coli* and has been widely used in clinical practice for over 40 years. L-ASNase monotherapy is an effective therapeutic approach to treat ALL, especially pediatric ALL (12, 66, 67). With the illustration of mechanisms of action, L-ASNase has become the cornerstone of multi-agent chemotherapy regimen for various malignancies (12). For example, combination chemotherapies containing L-ASNase have became the standard treatment for NK/T-cell lymphoma (68–70). And L-ASNase was part of chemotherapy regimens for pediatric AML in come clinical trails (71, 72).

Despite favorable efficacy, there are still several issues that require further attention. First, the toxicity profiles require improvement, which includes severe immunological side effects as well as non-immune-related toxicities such as pancreatitis, liver toxicities, venous thromboembolism (VTE), and neurotoxicity (12, 67). Moreover, with the increase of drug exposure in clinical practice, secondary drug resistances are gradually emerging. The resistance mechanism is generally believed to include the following aspects (12). ASNS expression is upregulated up to 7-fold by tumor cells to develop resistance to ASNase in Asn-depleted environment (73). Bone marrow-derived mesenchymal stromal cells (MSCs) may secrete L-asparagine and rescue leukemic blasts from L-ASNase deprivation. As a foreign protein, production of autoantibodies accelerates clearance of L-ASNase (74). Besides, some previously unknown mechanisms of L-ASNase resistance have been gradually perceived and recognized. Huntingtin associated protein 1 (HAP1) has been identified as a biomarker for L-ASNase resistance in ALL (75). HAP1 loss suppresses formation of the ternary complex that mediates endoplasmic reticulum (ER) Ca^2+^ release, thus preventing cell apoptosis induced by L-ASNase (75). In extranodal NK/T-cell lymphoma, brain cytoplasmic RNA 1 (BCYRN1), a long non-coding RNA (lncRNA), induced the degradation of p53, promoted autophagy and counteracted the effects of Asn deprivation, resulting in L-ASNase resistance (76).

To address the above issues, multiple novel L-ASNase formulations have been developed. PEGylated L-ASNase was produced by covalently conjugating monomethoxypolyethyleneglycol (PEG) to *E. coli* L-ASNase. The PEGylated formulation reduced the immunogenicity and prolonged the half-life period (77). But the antibodies induced by native *E. coli* L-ASNase might cross-react with PEGylated L-ASNase. Erwinia chrysanthemi L-ASNase was a solution to *E. coli* L-ASNase. It might induce less complications and toxicities (like coagulation abnormalities, neurotoxicity, and pancreatitis) (12).

### 2.4. Cysteine

As a semiessential AA, Cys is indispensable to various reactions, including GSH generation, LSC maintenance, and redox homeostasis. Although Cys could be compensatively generated from methionine (Met), the overall demand could not be met. Furthermore, leukemic cells and lymphoma cells displayed translationally silenced Cys synthetic enzymes, making them more vulnerable to Cys starvation than normal cells (78).

The impact of Cys on hematologic malignancies could be divided into two aspects, Cys metabolism and Cys transport. Regarding Cys metabolism, the LSCs of AML were highly dependent on Cys metabolism in order to fuel OXPHOS. Although cancer cells have been regarded as highly dependent on glycolysis instead of OXPHOS to produce energy, as proposed by Warburg effect, various cancer stem cells have been reported to rely more on OXPHOS than glycolysis, including AML and melanoma (79). The *de novo* AML LSCs have been proved heavily dependent on Cys metabolism and other AA metabolism in order to conduct OXPHOS and to survive (80). Cys was transformed to GSH, which was significantly reduced upon Cys depletion. The lack of GSH inhibited electron transport chain complex (ETC) II, which in turn damaged OXPHOS and caused death of LSCs. The killing effect was demonstrated specific to LSCs, without affecting normal hematopoietic stem and progenitor cells (HSPCs) (81). Venetoclax combined with azacitidine could also impair AA uptake and reduce LSCs (80).

Besides the shortage of GSH, Cys depletion has been demonstrated to activate 5′ adenosine monophosphate-activated protein kinase (AMPK) pathway, which in turn triggered autophagy. Moreover, mTORC1, which negatively regulated autophagy, was downregulated upon Cys depletion (82).

Regarding Cys transport, the *SLC7A11* gene encoded the transporter xCT, which imported Cys into cytoplasm while exported glutamate outside the cells. Since tumor cells exhibited higher ROS levels due to genetic or epigenetic change and aberrantly high metabolic activity, they required higher levels of reducing equivalents to repress the increased ROS, where the GSH made a great contribution to the reduction reaction. Since Cys was the component of GSH, tumor cells had a greater demand for Cys to generate sufficient GSH (11). Several studies have demonstrated increased xCT expression upon the surface of chemo-resistant tumor cells (83). Studies have found that *SLC7A11* mutation was an independent risk factor of survival of AML patients (84). Thus, xCT could be a promising target for cancer therapy, especially when combined with other chemotherapy which increased ROS of tumor cells.

Based on the impact of Cys on hematologic malignancies, innovative treatment of hematologic malignancies targeting Cys has been investigated. In terms of Cys depletion, a PEG-engineered cyst(e)inase enzyme, which stably depleted extracellular L-Cys and L-cystine, significantly decreased GSH and increased ROS, leading to cell cycle arrest and apoptosis of various tumor cells (85). It doubled the survival of the aggressive CLL TCL1-Tg:p53−/− mice, and selectively eliminated AML LSCs without influence on survival or colony forming ability of normal HSPCs (80). This enzyme was reported as safe and irreversible, since concentrations of L-Cys and L-cystine returned to untreated level subsequent to two to 4 days, and no obvious side effects were found in mice models (85).

Regarding Cys transport inhibition, several agents have been demonstrated to repress xCT function. Sulfasalazine, though previously developed to treat inflammation, has been reported to selectively inhibit xCT, decrease GSH, increase ROS, decrease proliferation and stimulate apoptosis of primary effusion lymphoma (PEL) cells (86). Sulfasalazine also reduced progression in PEL mice models (87). Pardieu et al. demonstrated that sulfasalazine inhibited xCT and caused oxidative stress-dependent leukemic cell death in primary samples of AML patients. In AML patients with *NPM1* mutations, Cys metabolism was upregulated with stronger cysteine dependency. Moreover, they found that sulfasalazine showed significant synergistic anti-leukemic effect with daunorubicin-cytarabine treatment in *NPM1*-mutated AML samples and patient-derived xenograft models (84). Erastin, though previously proved to trigger ferroptosis, has been recently found to inhibit xCT (88, 89). Erastin increased ROS and repressed viability of various solid tumor cells and AML cells (90–92). Sorafenib, a multi-receptor tyrosine kinase inhibitor, was also found to repress xCT function and stimulate ferroptosis (90). However, more investigation into the exact interaction between xCT function and ferroptosis in Cys starvation is needed to clarify the phenomenon.

Besides the Cys starvation to aggregate ROS and eliminate the existing tumor, Cys supplement has been reported to reduce ROS and delay tumor initiation. The N-acetyl-L-Cys (NAC) has been demonstrated to decrease ROS, repair DNA damage, and activate antioxidant enzymes in leukemic cells. Moreover, NAC could reduce leukemia initiation and organ damage, and prolonged survival in WEHI-3 leukemia mice models (93). Moreover, lymphoma incidence was relatively high in ataxia telangiectasia (AT) patients. Reliene et al. constructed an AT mice model and discovered that consistent dietary NAC supplement significantly decreased lymphoma incidence (94). However, the influence of NAC on tumor cells was controversial. Yedjou et al. aimed to investigate whether NAC supplement could reduce arsenic trioxide (ATO)-related toxicity in acute promyelocytic leukemia (APL) treatment. They discovered that the addition of NAC impeded ATO cytotoxicity, indicating the combination treatment of NAC with ATO was inappropriate to treat APL (95).

NAC has been widely used as antioxidants, and various studies have demonstrated that NAC could assist bone marrow reconstruction by promoting the function of bone marrow endothelial progenitor cells, especially for patients after allo-hematopoietic stem cell transplantation (HSCT) (96–98). A phase III, randomized, open-label trial was conducted in patients who received haploidentical HSCT. Patients underwent evaluation of endothelial cell proportion 2 weeks before conditioning treatment. Patients with endothelial cells less than 0.1% were recognized as high-risk, and were randomized into NAC prophylaxis group and non-prophylaxis group. Patients with endothelial cells more than 0.1% were considered as low-risk, and did not receive NAC prophylaxis. At 60 days after transplantation, the high-risk NAC prophylaxis group displayed significantly reduced poor graft function (PGF) and prolonged isolated thrombocytopenia (PIT) rate compared to high-risk non-prophylaxis group. Furthermore, the PGF and PIT rate was even lower in high-risk NAC prophylaxis group than low-risk group, indicating that NAC prophylaxis could overcome the disadvantage of poor endothelia cell function before transplantation (99).

### 2.5. Methionine

Met is an essential AA, and serves as the building block of Cys as well as polyamine. The demand for Met relies heavily on dietary supply, and the only way to compensatively generate Met is the Met salvage pathway. In the salvage pathway of normal cells, 5,10-methylene-THF was transformed into 5-methyl-THF *via* methylenetetrahydrofolate reductase (MTHFR). And the 5-methyl-THF was later generated into Met *via* methionine synthetase (MS) (100). However, in the salvage pathway of tumor cells, function of these crucial enzymes is frequently inhibited, rendering tumor cells significantly dependent on exogeneous Met, making Met as a promising anti-cancer target and labeled Met as a potential tracer for PET/CT.

The innovative application of ^11^C-MET PET/CT to diagnosis and staging has been developed recently. Myeloma cells had a high demand for Met in order to generate immunoglobulin, and ^11^C-MET PET/CT showed higher sensitivity than ^18^F-FDG PET/CT. Various studies have demonstrated that ^11^C-MET PET/CT was more sensitive to detect focal lesions and extramedullary disease. The SUV_mean_ of L2-L4 of ^11^C-MET PET/CT was significantly related to bone marrow plasma cell proportions, while that of ^18^F-FDG PET/CT showed no significant relationship between plasma cell proportion (101–103). Furthermore, studies have proved that higher total metabolic tumor volume and total lesion MET uptake were significant risk factors of PFS (104).

In lymphoma, the efficacy of ^11^C-MET PET/CT varies, depending on the site of lesions and the subtype of lymphoma. In CNS detection, ^11^C-MET PET/CT seemed superior to ^18^F-FDG PET/CT in differentiation diagnosis of CNS lymphoma from glioblastoma multiformes (GBMs). A study enrolled seven DLBCL and 15 GBM patients. ^11^C-MET PET/CT performed better than ^18^F-FDG PET/CT by completely differentiating DLBCL from GBM based on ΔSUV_max_ < 1.17, with no false negativity or false positivity (105). Furthermore, in primary CNS lymphoma patients, higher tumor-to-normal ratio of ^11^C-MET PET/CT was significantly related to worse PFS, while that of ^18^F-FDG PET/CT showed no significant relationship to prognosis (106). However, other studies claimed that ^11^C-MET PET/CT failed to differentiate primary CNS lymphoma or GBMs (107). In abdomen detection, ^11^C-MET PET/CT could not effectively differentiate the physiologic high Met uptake or malignant high uptake in pancreas and liver, limiting its application to abdomen detection (106).

Met is also indispensable to epigenetic regulation, because the S-adenosylmethionine (SAM) in the Met cycle serves as the only source to provide methyl residues to DNA and histone methylation. However, tumor cells displayed higher demand for Met and aberrantly stimulated Met adenosyltransferase (MAT), upstream of the SAM (108). The methyltransferase action was in turn overstimulated, which converted SAM mostly into S-adenosylhomoCys (SAH), leading to insufficient methyl residues from SAM and inability to methylate DNA or histone, interfering with the epigenetic regulation. For example, MLL-rearranged leukemia has been demonstrated to rely on the H3K79 methyltransferase DOTL1, and this disease entity was characterized as high demand for methyl residues to ensure DOTL1 function. Barve et al. explored whether inference with Met/SAM metabolism could inhibit MLL-rearranged leukemia. Not only Met depletion but also SAM inhibitor 3-deazaadenosine could decrease methylation level, inhibit proliferation and stimulate apoptosis of the MLL leukemic cells. Moreover, 3-deazaadenosine showed synergistic effect with 5 + 3 induction chemotherapy and extended survival of MLL-rearranged leukemia mice models (109). Dietary Met starvation has been demonstrated to impair H3K36me3, decrease total RNA concentration, stimulate apoptosis and cell cycle arrest of AML cells, delay AML progression in mice models without obvious adverse effects (110, 111). Thus, MAT inhibition might serve as a promising approach to treat hematologic malignancies with epigenetic alteration or IDH/TET mutations. SAM decarboxylase inhibitors have been explored in cancer treatment. SAM486A is an innovative second-generation SAM decarboxylase inhibitor, which has been tested in a phase II, multicenter clinical trial. The study enrolled 41 relapsed or refractory NHL patients, who received SAM486A monotherapy for eight cycles or until disease progression. The ORR was 18.9% with a tolerable safety profile (112). AG-270, a first-in-class MAT2A inhibitor which effectively decreased SAM and impeded tumor cell proliferation, is being investigated in lymphoma and solid tumor patients in an ongoing phase I trial (113).

Single nucleotide polymorphisms in various Met metabolic enzymes, including MS A2765G, MTRR A66G, MTHFR 677 or 1,298 gene polymorphism displayed different susceptibility to lymphoma, ALL, AML, and Chronic myeloid leukemia (CML), probably due to subtypes of malignancies, ethic factors, and gender (114–119).

### 2.6. Serine

Serine is a non-essential AA and serves as building blocks of glycine, phospholipids, and nucleotides. Tumor cells upregulate serine-related enzymes, including PHGDH, PSPH, and PSAT1, in order to meet the increased serine demand, indicating these enzymes as druggable target to treat hematologic malignancies. In *FLT3*-ITD-driven AML, *FLT3*-ITD upregulated serine synthesis *via* ATF4 (120). In T-ALL, PSPH transcription and translation were significantly increased in T-ALL cell lines and primary bone marrow cells of T-ALL patients. PSPH upregulation led to increase of serine synthesis, which not only directly promoted leukemic cell proliferation through increased purine and folate, but also indirectly promoted stromal cell proliferation through increased glycine (121). In MM, serine upregulation was associated with bortezomib resistance (122). These findings above indicated that serine metabolic enzymes could serve as promising target to treat hematologic malignancies.

WQ-2101 is a PHGDH inhibitor, and has been evaluated in *FLT3*-ITD-driven AML. Both WQ-2101 and PHGDH knockout could repress proliferation of leukemic cells *in vitro* and *in vivo*. Furthermore, WQ-2101 could increase the extent of DNA damage caused by cytarabine, increasing the sensitivity of leukemic cells to cytarabine (120). In T-ALL, inhibition of PSPH by shPSPH could repress proliferation and trigger apoptosis of leukemic cells *in vitro*. It could also alleviate splenomegaly and decrease leukemic cells in the bone marrow and spleen of T-ALL mice models (121).

### 2.7. Branched chain amino acids

Branched chain AAs (BCAAs) include valine, leucine, and isoleucine, and have been found related to aggressiveness of leukemia. BCAA transaminase 1 (BCAT1) and BCAA transaminase 2 (BCAT2) produce BCAAs *via* aminating branched chain keto acids. It has been reported that BCAT1 was overexpressed in CML patients and mouse models, and was related to worse prognosis. The oncogenic protein Musashi2 bound to BCAT1 and upregulate its expression, leading to progression of blast crisis CML. Inhibition of BCAT1 expression stimulated differentiation of blast cells and repressed blast crisis CML (123). Furthermore, BCAT1 and BCAT2 were upregulated by the overexpressed oncogenic m^6^A methyltransferase, METTL16, leading to BCAA reprogram in LSCs and leukemia-initiating cells. METTL16 knockout significantly repressed AML leukemogenesis and impeded LSC self-renewal (124). Studies have explored the BCAA-related metabolism in CD34+ cells of healthy controls, AML patients and ALL patients. The BCAA transporters, BCAT, BCAA concentration and α-KG were significantly higher in AML and ALL than those in healthy controls. Dietary BCAA starvation significantly repressed proliferation, development and self-renewal of LSCs in xenogeneic transplantation models (125). In a word, BCAA reprogram is relatively common in leukemia, which might maintain LSC stemness and promote leukemia transformation. BCAA starvation might be a promising strategy to postpone leukemogenesis.

## 3. Challenges and opportunities

### 3.1. Potential immunotoxicity

Although AA starvation could impair viability of tumor cells, they could also possibly impair the function of immune cells, leading to immune escape. For example, since Met serves as the major source of methyl residue for DNA and histone methylation, starvation of Met inevitably caused epigenetic malfunction and impaired T cell function. To avoid the influence of Met starvation on normal cells, supplement of homocysteine, vitamin B12 and folate might assist Met salvage pathway in normal cells. Furthermore, cystine played an important role in T cell expansion as well as activation. Although Cys starvation has been reported effective to treat hematologic malignancies, cysteinase was also reported to promote anti-tumor immune response. The impact of AA starvation on immune cells has been controversial and requires further investigation to reveal their interaction.

### 3.2. Insufficient efficacy/drug resistance-combination therapy

Studies have found that chemoresistance is frequently related to the protection of tumor cells by the AA-providing bone marrow stromal cells. For example, Ede et al. explored the mechanism of chemoresistance to parthenolide in pediatric T-ALL patients. They discovered that the bone marrow mesenchymal stromal cells generated and provided Cys to leukemic cells, rendering them survival benefits (126). Moreover, in CLL, resistance to ASNase was attributable to the cystine provided to tumor cells by stromal cells (127). Thus, combination treatment of AA starvation with conventional chemotherapy might overcome chemoresistance and achieve a higher response rate.

### 3.3. Limited half-life period

Although AA starvation has shown some effect on treating hematologic malignancies, the half-life period of starvation was sometimes short in its current form, limiting its efficacy and feasibility in the future clinical practice. For example, the Cys returned to previous level only 2 days after injection of a cyst(e)inase into mice models, requiring improvement of its form to ensure more stable and sustainable effect *in vivo*^[64]^.

## 4. Conclusion

AAs are indispensable to development and progression of hematologic malignancies, with tumor cells demanding significantly larger amount of various AAs for enhanced proliferation, abnormal signaling transduction, and epigenetic dysregulation. The innovative therapy targeting AAs mainly aims at AA metabolism (either starvation or supplement) and transportation. Numbers of AA starvation therapy has been approved for hematologic treatment, such as L-ASNase. Treatment aiming at AA supplement, such as NAC, or transportation, such as xCT inhibitors, is being investigated in clinical trials and showing encouraging results. The innovative AA therapy will provide a promising approach to further improve treatment of hematologic malignancies, especially when combined to traditional chemotherapy in a more stable form with longer half-life period.

## Author contributions

MW: writing—original draft, writing—review and editing. AZ: writing—original draft, writing—review and editing. ML: writing—original draft. TN: validation, writing—review and editing. All authors contributed to the article and approved the submitted version.

## Funding

This work was supported by Incubation Program for Clinical Trials (No. 19HXFH030), Achievement Transformation Project (No. CGZH21001), 1.3.5 Project for Disciplines of Excellence, West China Hospital, Sichuan University (No. ZYJC21007), Translational Research Grant of NCRCH (No. 2021WWB03), Chengdu Science and Technology Program (No. 2022-YF05-01444-SN), Key Research and Development Program of Sichuan Province (No. 2023YFS0031), and National Key Research and Development Program of China (No. 2022YFC2502603).

## Conflict of interest

The authors declare that the research was conducted in the absence of any commercial or financial relationships that could be construed as a potential conflict of interest.

## Publisher’s note

All claims expressed in this article are solely those of the authors and do not necessarily represent those of their affiliated organizations, or those of the publisher, the editors and the reviewers. Any product that may be evaluated in this article, or claim that may be made by its manufacturer, is not guaranteed or endorsed by the publisher.
